# Prevalence and determinants of *Leishmania major* infection in emerging and old foci in Tunisia

**DOI:** 10.1186/1756-3305-7-386

**Published:** 2014-08-20

**Authors:** Jihene Bettaieb, Amine Toumi, Sadok Chlif, Bilel Chelghaf, Aicha Boukthir, Adel Gharbi, Afif Ben Salah

**Affiliations:** Department of Medical Epidemiology, Pasteur Institute of Tunis, Laboratory of Transmission, Control and Immunobiology of Infections (LR11IPT02), 13 Place Pasteur BP-74, 1002 Tunis Belvedere, Tunisia

**Keywords:** Zoonotic Cutaneous Leishmaniasis, Leishmanin Skin Test, Prevalence, Risk factors, Tunisia

## Abstract

**Background:**

Zoonotic Cutaneous Leishmaniasis (ZCL) due to *Leishmania major (L. major*) is still a serious public health problem in Tunisia. This study aimed to compare the prevalence and risk factors associated with *L. major* infection in old and new foci using leishmanin skin test (LST) in central Tunisia.

**Methods:**

A cross sectional household survey was carried out between January and May 2009 on a sample of 2686 healthy individuals aged between 5 and 65 years. We determined the prevalence of *L. major* infection using the LST. Risk factors of LST positivity were assessed using a logistic regression model.

**Results:**

The overall prevalence of LST positivity was 57% (95% CI: 53–59). The prevalence of *L. major* infection was significantly higher in the old focus (99%; 95% CI: 98–100) than in the emerging foci (43%; 95% CI: 39–46) (p = <0.001). Multivariate analysis of LST positivity risk factors showed that age, the nature of the foci (old/emerging), personal and family history of ZCL are determinants of positive LST results.

**Conclusion:**

The results updated the current epidemiologic profile of ZLC in central Tunisia. Past history of transmission in a population should be considered as a potential confounder in future clinical trials for drugs and vaccines against *L. major* cutaneous leishmaniasis.

## Background

Cutaneous leishmaniasis (CL) due to *Leishmania major (L. major*) is still a serious public health issue in North African countries [1]. Previous studies showed that asymptomatic infection with *L. major* may occur in endemic areas but the extent of this phenomenon has not been fully evaluated [2]. People without patent disease may show evidence of infection as demonstrated by a positive Leishmanin skin test (LST). The test is currently used to measure the prevalence of *Leishmania* exposure in human communities and was considered as an important tool for epidemiological surveys of leishmaniasis transmission [3–6]. The epidemiological significance of a positive LST reaction has been described elsewhere [7–14].

It is widely accepted that the leishmaniases are dynamic diseases and the circumstances of transmission are continually changing in relation to environmental, demographic and human behavioural factors [15]. In Tunisia, CL is caused by *L. major* and transmitted by *Phlebotomus papatasi*[16]. Rodents constitute the reservoir for CL, herein called zoonotic cutaneous leishmaniasis (ZCL) [16–21]. The epidemic of CL emerged in central Tunisia in 1982 and expanded to the whole central and southern parts of the country (15/23 governorates are considered as endemic in 2002). The epidemics are cyclic and annual incidence ranges from 2000 to 10000 cases. In the recent decade, several new foci have been reported indicating the potential spread of disease in Tunisia [22]. The epidemiological features of CL in emerging foci are poorly documented. In addition, the risk factors associated with the transmission and the extension of the disease were not clearly elucidated. Features and significance of LST positive reaction among humans might be variable in old foci, where the immune system has been continuously challenged by bites of infectious flies, and newly emerging ones where exposure is more recent and short. Reliable population-based prevalence data are therefore essential for understanding the importance of the problem for planning, monitoring and evaluating leishmaniasis control programmes. To our knowledge, this is the first study attempting to compare LST in old and emerging *L. major* foci.

The aim of this study is to estimate the prevalence and risk factors associated with LST reactivity in old and emerging ZCL foci in central Tunisia as an indicator of the cumulative leishmanial exposure experienced by the community. Findings will support control strategies and fine tune the methods of clinical trials of anti-leishmanial drugs and vaccines.

## Methods

### Study area

**Figure 1:**
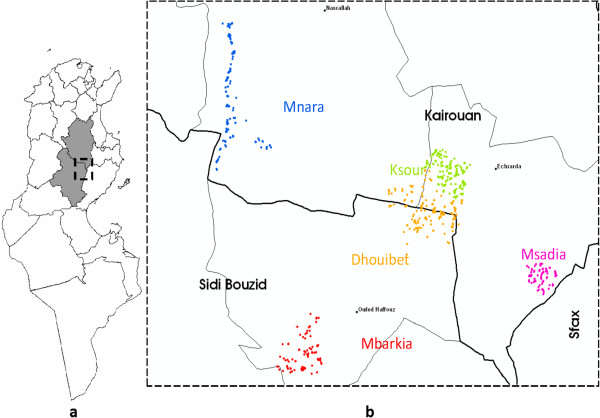
**Spatial distribution of dwellings included in the study. (a)** Kairouan and Sidi Bouzid Governorates location within Tunisia. **(b)** Location of study area within Kairouan and Sidi Bouzid Governorates. Dwellings included randomly in the study were located by Global Positioning System (GPS) (a dot represents a dwelling).

The study was conducted in an endemic area of CL situated in central Tunisia in two governorates, Sidi Bouzid (35°02'00''N, 9°30'00''W) and Kairouan (35°40'00''N, 10°06'00''W) with an overall area of 13,706 km^2^ (Figure 1). The governorates share the same topography and climate. The study area is located in the arid zone of Tunisia, a climatic transition between the Mediterranean zone and the Sahara region. Most of the study population resided in rural communities.

### Selection of study population

A two stage cluster sampling scheme with clusters of equal sizes was applied to randomly include 2800 individuals from the study area. The first stage consisted of a random selection of 25 districts (each district contains about 70 dwellings in general) from five villages: Mbarkia and Dhouibet from Sidi Bouzid, Mnara, Msaadia and Ksour from Kairouan (Figure 1). The choice of these villages was made especially with regard to nature of the foci (old versus emerging). Indeed, Mnara constitued an important CL old-focus of *L. major* in this region where, Mbarkia, Dhouibet Msaâdia and Ksour are considered as emerging foci on the basis of case notification data in the district epidemiological surveillance system. The second stage consisted of a random selection of ~25 to 30 dwellings per district to permit a sub sample of 112 volunteers per district.

All individuals aged between 5 and 65 years in the selected dwellings who gave their written informed consent (or their parents or legal guardians consent in case of minors) were enrolled. Individuals with serious concomitant disease as identified by the medical history and children less than 5 years of age were not eligible for ethical reasons.

### Study design and data collection

A cross sectional household survey was carried out between January and May 2009. The eligible subjects were interviewed by trained local interviewers by house to house visit. Standardized questionnaires, which sought specific information regarding socio-demographic characteristics, behaviors, occupational activities, level of income, past history of ZCL, and household characteristics were completed. For each volunteer, the skin was thoroughly examined for the detection of typical scars. A LST was performed for volunteers to assess exposure to *L. major* infection.

### Leishmanin Skin TEST (LST)

The antigen used in the LST was obtained from The Pasteur Institute of Iran prepared from Iranian *L. major* strains. The skin test was performed by intradermal injection in the inner surface of the forearm of 0.1 ml of leishmanin (suspension containing 5×10^6^ killed promastigotes of *L. major* in 0.5% phenol saline). Readings were taken 48 to 72 hours later using the ballpoint-pen technique of Sokal to determine the 2 diameters of the induration [23]. The LST result was considered positive if the mean of the two measurements was five mm or more [5, 14, 24, 25].

### Statistical analysis

Data were weighted to adjust for sampling probability selection and non-response rate. Direct standardised prevalence rates were performed to account for differences in the age structures among villages. The reference population was obtained from the last census data (2004) of the study population.

Proportions were compared by *χ*^2^ test. We compared the age distribution and the size of the response for the *LST* reaction between villages using Kruskal-Wallis rank test followed by Dunn's multiple comparison tests.

Risk factors for positive LST results (LST^+^) were assessed in a univariate and mulitivariate logistic regression analysis for new and old foci separately and for the whole survey. Variables included in the univariate analysis were age groups, gender, family size (<6 et ≥6), shelter for animals, well for irrigation, cemented walls, number of rooms in the house, yearly income, farming occupation, personal past history of ZCL, presence of scars and history of ZCL among family members (number of past cases). Variables associated with LST^+^ at the p < 0.25 level in the univariate analysis were incorporated in a weighted binary logistic regression procedure. The final model was obtained by a backward selection strategy. P values ≤ 0.05 were considered statistically significant.

Statistical analyses were performed using STATA/IC 11.0 (StataCorp, College Station, TX).

### Ethics statement

The study was approved by the ethical committee of The Pasteur Institute of Tunis.

## Results

A total of 2686 individuals of both sexes and different age groups were enrolled from the 5 villages. Two thousand and one hundred individuals (78%) were skin tested. Reading of the indurations was carried out for 77% of persons enrolled (2079/2686). Most of the non- participants were absent during household visits.

Weighted demographic and socioeconomic information of the study participants are presented in Table 1. The median (IQR) age of all study participants was 25 (13–43) years, nearly 52% of them were female. Approximately 3% of the enrolled had at least some high school education and 89% had a yearly income under 1.5 thousand USD. Median (IQR) family size was 6 (5–8) persons. About 45% of dwellings had a well for irrigation and only 3% of subjects had a house with cemented walls. Almost all households had animal shelters (98%).

**Table 1:** **Demographic and socioeconomic characteristics of study participants by village**

	Mnara	Mbarkia	Dhouibet	Msaadia	Ksour	Total
**Variables**	(n = 397)	(n = 266)	(n = 441)	(n = 348)	(n = 627)	(n = 2079)
**Median Age** (IRQ) (years)	20 (13–44)	26 (12–43)	25 (14–43)	26 (15–43)	24 (11–39)	25 (13–43)
**Females** (%)	53 (47–59)	51 (45–57)	56 (51–60)	56 (50–61)	50 (45–55)	52 (50–55)
**Education** (%)						
Under school age	2 (0–3)	3 (1–5)	3 (2–5)	2 (0–4)	4 (2–5)	3 (2–4)
Illiterate	21 (16–26)	8 (5–12)	15 (11–19)	16 (13–20)	18 (14–22)	16 (14–18)
Primary	45 (39–52)	53 (45–60)	53 (47–58)	52 (46–58)	55 (49–61)	52 (49–55)
Secondary	27 (21–32)	32 (25–39)	27 (22–32)	26 (20–32)	22 (17–27)	26 (24–29)
University	5 (1–9)	4 (1–8)	2 (0–3)	3 (1–5)	1 (0–2)	3 (2–4)
**Farming occupation** (%)	2 (0–4)	3 (1–6)	5 (2–7)	2 (0–4)	4 (2–6)	3 (2–4)
**Yearly income** (%)						
(1,000USD)						
< 1.5	91 (85–97)	74 (63–84)	89 (84–95)	85 (77–93)	98 (95–100)	89 (86–92)
≥ 1.5	8 (2–14)	26 (16–37)	11 (5–16)	15 (7–23)	3 (0–5)	11 (8–14)

IRQ: Interquartile range.

Note: data are percentage (95% Confidence Interval).

(Counts reflect weighting).

There were no significant differences between the study villages regarding demographic and socioeconomic characteristics of households. Of those who received the LST, 25% had personal history of ZCL (new foci: 18%; old focus: 38%; p < 0.001), while a higher proportion reported family history of ZCL (new foci: 49%; old focus: 72%; p < 0.001).

Table 2 shows the weighted and direct standardised prevalence rate of *L. major* infection for each village in the study area. The age standardised prevalence of LST positivity ranged from about 36% in Msaadia to 97% in Mnara, which represents the old focus of the study. There was a significant difference between the 5 villages in the prevalence rates (p < 0.001) and between new foci combined and the old one (p < 0.001). No difference was observed between the 4 villages considered as part of emerging foci (p = 0.3). The overall age standardised prevalence of LST positivity was 51%.

**Figure 2:**
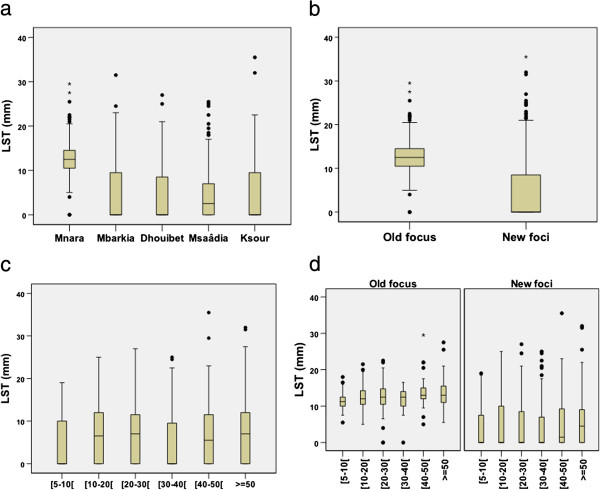
**Distribution of LST size reaction (a) by village, (b) by foci, (c) by age groups (d) by age groups and foci.**

Figure 2 shows the distribution of LST size reaction. The Kruskal-Wallis test revealed significant differences in the LST reactivity between villages (p = 0.001). According to Dunn's test, Mnara appeared to differ significantly from the other villages. In fact, the highest median size was observed in Mnara (12.5 mm) and ranged from 0 to 2.5 mm in the other villages.

**Table 2:** **Prevalence of leishmanin skin test positivity by village**

Village	N	Weighted prevalence	Standardised prevalence	Standardised weighted prevalence
		% (95% CI)	% (95% CI)	% (95% CI)
**New focus**				
Ksour	627	43 (37–48)	44 (39–50)	39 (35–43)
Mbarkia	266	47 (39–53)	44 (35–53)	44 (39–49)
Dhouibet	441	41 (35–46)	39 (33–44)	39 (34–44)
Msaadia	348	39 (31–44)	36 (30–43)	36 (30–43)
**New Foci combined**	1682	43 (39–46)	40 (37–44)	38 (36–40)
**Old focus**				
Mnara	397	99 (98–100)	97 (86–100)	99 (92–100)
**Total**	2079	57 (53–59)	51 (48–54)	55 (53–57)

N: number of subjects with reading skin test.

CI: Confidence Interval.

There was a statistically significant variation across age groups of the median of the LST reactivity among the total sample of tested persons (p < 0.001). The highest median size (7.5 mm) was seen in the age group > 50 years old. LST reactivity size remained strongly associated with age in each foci category (old focus; p = 0.0006 and new focus; p = 0.0041).

Table 3 shows adjusted odds ratio of the variables retained in the final logistic regression models. When we consider the final model for new foci, no association was found with gender and cemented wall house. Strong associations were identified between LST positivity and presence of personal as well as family history of ZCL. The risk increased with the number of family historical ZCL cases. Those who had 3 or more index cases had almost 3.1 times the risk of having positive LST response compared to those without family past history of ZCL. The influence of age on Leishmaniasis risk was significant. Adults over the age of 50 years had the highest risk.

**Table 3:** **Risk factors associated with LST positivity as a result of multiple logistic regression analysis**

	New foci		New and old foci combined	
**Covariates**	AOR* (95% CI)	P value	AOR* (95% CI)	P value
**Age** (5–10 years)	Reference	<0.001	Reference	<0.001
10-30 years	1.12 (0.79-1.58)		1.09 (0.77-1.54)	
30-40 years	1.80 (1.15-2.80)		1.78 (1.15-2.75)	
40-50 years	1.83 (1.19-2.81)		1.81 (1.19-2.77)	
≥ 50 years	2.38 (1.48-3.82)		2.38 (1.50-3.79)	
**Personal history of ZCL** (No)	Reference	<0.001	Reference	<0.001
Yes	3.58 (2.35-5.44)		3.35 (2.21-5.08)	
**Foci** (New)			Reference	
Old	NI	-	122.75 (39.71-379.48)	<0.001
**Family ZCL number of past cases** (0 case)	Reference	<0.001	Reference	<0.001
1 case	1.57 (1.15-2.14)		1.60 (1.18-2.17)	
2 cases	1.88 (1.19-2.97)		1.85 (1.17-2.91)	
≥ 3 cases	3.08 (1.79-5.30)		3.02 (1.77-5.16)	

*AOR: Adjusted Odds Ratio using the category between brackets as reference.

CI: Confidence Interval.

NI: Not included.

(Final models).

In combined foci analysis, the results were similar to those obtained with emerging foci. After adjustment, the risk of LST positivity increased by more than one hundred times in the old focus compared to the emerging ones.

## Discussion

In this paper we presented results of the first large LST-epidemiological study of foci with different longstanding *Leishmania* endemicity in central Tunisia (old and emerging ones). Our data demonstrated the hyperendemicity of this region and confirmed the initial observation of a significant higher prevalence of infection in the old focus. Lower but still high LST positive proportions were observed in emerging foci indicating active continuous ZCL transmission. Multiple logistic regression analyses showed significant associations between LST positivity and age, personal and family history of ZCL, and foci chronological emergence of Leishmaniasis in humans.

The overall prevalence of LST positive individuals was greater than prevalence data reported in previous surveys elsewhere in the country [22]. This may reflect the increasing putative endemicity of ZCL transmission in Tunisia over time and across geographic space, suggesting that the control strategy was not effective enough to reduce man vector contacts in endemic regions. Indeed, examination of the Tunisian surveillance system showed that control measures were limited to case notification, passive detection and treatment of ZCL cases. As such, it was therefore not possible to reduce the temporal and spatial spread of the disease.

The lack of efficacy of the control tools available is partly explained by the complexity of the transmission cycle and the insufficient knowledge of the epidemiology and the natural history of the disease. Consequently, an appropriate control programme has not been defined and implemented. For this purpose, our primary objective was identification of the potential risk factors associated with LST positivity as indicator of the human cumulative exposure to *L. major* infection.

In this setting, the major risk factor for LST positivity was the past history of transmission in a given geographic area. Several data published elsewhere have revealed that LST positivity increased with length of residence in endemic areas, as an indicator of time exposure to the parasite [26]. Our findings provide additional evidence of this and suggest that people who resided in the old focus acquired a relative protection due to the presence of continuous boost of the immune system by exposure to infectious sandfly bites as supported by the observed higher size of LST reaction. Moreover, the higher rate of infection in the old focus may be due to a higher density and infection rate of rodent reservoirs and, consequently, a higher infection rate of vector sandflies.

In established endemic areas, previous studies indicate that the positivity and the size of LST increase steadily with age, presumably because of parasite load and consequent acquired immunity among immunocompetent individuals [26]. Based on this, our primary hypothesis was that the LST positive reactions are more frequent among older age groups in old foci but remain the same among all age groups in emerging ones. Our findings support the former but not the latter hypothesis. Positivity and reactivity size of LST increased likewise with age in our study villages considered as emerging foci. However, because of the very close geographic proximity between emerging and old foci, a large number of villagers commute frequently between villages for socio-economic reasons. Besides, some villagers leave their families in the village and move and establish for several months in other areas in search of better work opportunities. Thus, we cannot exclude the possibility that some older household members could get infected with *Leishmania* elsewhere before returning to the surveyed village, which could have potentially confounded the study results. Furthermore, determinants of pathogenicity of *Leishmania* infection include the host’s innate susceptibility and acquired resistance, which is related to age [27, 28]. This in addition to virulence characteristics of the parasite strain, and sandfly behaviours [29].

No significant difference was found between males and females suggesting that they are equally exposed to infection. Likewise, Leishmaniasis risk was not associated with occupation or outdoor activities. Thus, transmission may not be related to behavioural patterns or other gender associated activities that might modify exposure to the vector as shown in previous studies [30–32]. In other settings where domestic transmission predominates, there was no relationship regarding gender and positivity of LST [33], which is consistent with the present results. Presence of active rodent burrows and trapping of infected sandflies in the surroundings of houses will support this hypothesis.

Leishmanin test positivity was associated with previous personal history of ZCL confirming previous reports that following a symptomatic infection, an individual acquires a certain form of resistance but this resistance is neither absolute nor lifelong [28]. Prospective population based studies will elucidate the immune correlates for protection to better characterize resistant individuals.

A person’s probability of having a positive LST was strongly associated with the presence of family history of disease and increased significantly with the number of past ZCL cases among other persons in the same household. This finding indicates a significant clustering of ZCL transmission within households. However environmental conditions of the household and income, do not appear to be significant determinants of LST positivity. But, our sample was drawn from populations with homogenous socio-economic status profiles, which could limit our ability to find any significant association between house characteristics and LST positive risk.

*Leishmania* infection has traditionally been found to be associated with working in or near farms in Tunisia [22]. Natural and man-made environmental changes in land coverage and use, shifts to other types of farming, and unplanned human settlements, have probably shifted the risk of infection from sylvatic areas to rural settlements as suggested by the current work [34]. The increase in the number of ZCL cases in recent years could be related to the increase of domestic transmission cycle, previously unknown, which presents new opportunities for control options. Regular trapping of sandflies at representative sites should be used to monitor the spatial distribution of vector abundance and thus, populations at risk so as to implement control strategies based on evidence. Means to reduce vector exposure in and around houses have a major impact in reducing the risk of domestic ZCL transmission in endemic areas [29, 35–37].

In this study, we used LST as a marker of previous exposure to *L. major*. LST is proved to be a good screening and epidemiological tool for identification of ongoing *Leishmania* transmission irrespective of disease presentation and has been used in many epidemiologic population based surveys [3–6]. Nevertheless, the LST has its limitations in CL, some are related to the level of sensitivity of the Leishmanin used [38–40]. Different sources of *Leishmania* antigen derived from different strains are used, which makes the comparison between studies difficult. The Leishmanin antigen used here was obtained from The Pasteur Institute of Iran prepared from Iranian *L. major* strains. If the LST antigen could be prepared using local strains, this might possibly increase its sensitivity in persons with a previous ZCL history, which is crucial to more accurately determine the extent of exposure as implied by other researchers [41]. Thus, a standardized skin-test that uses antigens derived from local strains should be developed for future use in future epidemiological studies in Tunisia.

## Conclusions

The present work updated the current epidemiologic profile of ZCL in central Tunisia by assessing the burden of exposure and determinants of *L. major* infection. We confirmed the hyperendemicity of this region and the increasing domestication of ZCL transmission. While posing new challenges, this process raises hopes for the effectiveness of interventions that reduce human sandfly contact inside and around houses. Due to its limited health resources, prioritization of successful public health interventions and identification of populations likely to be exposed to sandflies are essential in disease management in Tunisia.

## References

[1] Hotez PJ, Savioli L, Fenwick A (2012). Neglected tropical diseases of the Middle East and North Africa: review of their prevalence, distribution, and opportunities for control. PLoS Negl Trop Dis.

[2] Sassi A, Louzir H, Ben Salah A, Mokni M, Ben Osman A, Dellagi K (1999). Leishmanin skin test lymphoproliferative responses and cytokine production after symptomatic or asymptomatic Leishmania major infection in Tunisia. Clin Exp Immunol.

[3] Peters W, Killick-Kendrick R, PEC M-B (1987). Diagnosis. The leishmaniasis in biology and medicine, clinical aspects and control.

[4] Alvarado R, Enk C, Jaber K, Schnur L, Frankenburg S (1989). Delayed-type hypersensitivity and lymphocyte proliferation in response to Leishmania major infection in a group of children in Jericho. Trans R Soc Trop Med Hyg.

[5] Weigle KA, Valderrama L, Arias AL, Santrich C, Saravia NG (1991). Leishmanin skin test standardization and evaluation of safety, dose, storage, longevity of reaction and sensitization. Am J Trop Med Hyg.

[6] Weigle KA, Santrich C, Martinez F, Valderrama L, Saravia NG (1993). Epidemiology of cutaneous leishmaniasis in Colombia: a longitudinal study of the natural history, prevalence, and incidence of infection and clinical manifestations. J Infect Dis.

[7] Ali A, Ashford RW (1993). Visceral leishmaniasis in Ethiopia. II. Annual leishmanin transformation in a population. Is positive leishmanin reaction a life-long phenomenon?. Ann Trop Med Parasitol.

[8] Ali A, Ashford RW (1993). Visceral leishmaniasis in Ethiopia. I. Cross-sectional leishmanin skin test in an endemic locality. Ann Trop Med Parasitol.

[9] Bettini S, Gramiccia M, Gradoni L, Pozio E, Mugnai S, Maroli M (1983). Leishmaniasis in Tuscany (Italy): VIII. Human population response to leishmanin in the focus of Monte Argentario (Grosseto) and epidemiological evaluation. Ann Parasitol Hum Comp.

[10] Davies CR, Llanos-Cuentas EA, Pyke SD, Dye C (1995). Cutaneous leishmaniasis in the Peruvian Andes: an epidemiological study of infection and immunity. Epidemiol Infect.

[11] Gramiccia M, Bettini S, Gradoni L, Ciarmoli P, Verrilli ML, Loddo S, Cicalo C (1990). Leishmaniasis in Sardinia. 5. Leishmanin reaction in the human population of a focus of low endemicity of canine leishmaniasis. Trans R Soc Trop Med Hyg.

[12] Marty P, Le Fichoux Y, Giordana D, Brugnetti A (1992). Leishmanin reaction in the human population of a highly endemic focus of canine leishmaniasis in Alpes-Maritimes, France. Trans R Soc Trop Med Hyg.

[13] Nandy A, Neogy AB, Chowdhury AB (1987). Leishmanin test survey in an endemic village of Indian kala-azar near Calcutta. Ann Trop Med Parasitol.

[14] Pampiglione S, Manson-Bahr PE, La Placa M, Borgatti MA, Musumeci S (1975). Studies in Mediterranean leishmaniasis. 3. The leishmanin skin test in kala-azar. Trans R Soc Trop Med Hyg.

[15] Toumi A, Chlif S, Bettaieb J, Ben Alaya N, Boukthir A, Ahmadi ZE, Ben Salah A (2012). Temporal dynamics and impact of climate factors on the incidence of zoonotic cutaneous leishmaniasis in central Tunisia. PLoS Negl Trop Dis.

[16] Aoun K, Bouratbine A (2014). Cutaneous leishmaniasis in North Africa: a review. Parasite.

[17] Cross ER, Newcomb WW, Tucker CJ (1996). Use of weather data and remote sensing to predict the geographic and seasonal distribution of Phlebotomus papatasi in southwest Asia. Am J Trop Med Hyg.

[18] Colacicco-Mayhugh MG, Masuoka PM, Grieco JP (2010). Ecological niche model of Phlebotomus alexandri and P. papatasi (Diptera: Psychodidae) in the Middle East. Int J Health Geogr.

[19] Mostafa BH, Abderrazak Souha B, Sabeh F, Noureddine C, Riadh BI (2006). Evidence for the existence of two distinct species: Psammomys obesus and Psammomys vexillaris within the sand rats (Rodentia, Gerbillinae), reservoirs of cutaneous leishmaniasis in Tunisia. Infect Genet Evol.

[20] Fichet-Calvet E, Jomaa I, Ben Ismail R, Ashford RW (2003). Leishmania major infection in the fat sand rat Psammomys obesus in Tunisia: interaction of host and parasite populations. Ann Trop Med Parasitol.

[21] Mott KE, Nuttall I, Desjeux P, Cattand P (1995). New geographical approaches to control of some parasitic zoonoses. Bull World Health Organ.

[22] Salah AB, Kamarianakis Y, Chlif S, Alaya NB, Prastacos P (2007). Zoonotic cutaneous leishmaniasis in central Tunisia: spatio temporal dynamics. Int J Epidemiol.

[23] Sokal JE (1975). Editorial: Measurement of delayed skin-test responses. N Engl J Med.

[24] Acedo Sanchez C, Martin Sanchez J, Velez Bernal ID, Sanchis Marin MC, Louassini M, Maldonado JA, Morillas Marquez F (1996). Leishmaniasis eco-epidemiology in the Alpujarra region (Granada Province, southern Spain). Int J Parasitol.

[25] Badaro R, Jones TC, Lorenco R, Cerf BJ, Sampaio D, Carvalho EM, Rocha H, Teixeira R, Johnson WD (1986). A prospective study of visceral leishmaniasis in an endemic area of Brazil. J Infect Dis.

[26] Moral L, Rubio EM, Moya M (2002). A leishmanin skin test survey in the human population of l'Alacanti region (Spain): implications for the epidemiology of Leishmania infantum infection in southern Europe. Trans R Soc Trop Med Hyg.

[27] Strelkova MV (1991). Susceptibility to and the characteristics of the course of experimental leishmaniasis in different species of mammals infected with Leishmania major, L. turanica and L. gerbilli. Med Parazitol (Mosk).

[28] Ben Salah A, Louzir H, Chlif S, Mokni M, Zaatour A, Raouene M, Ismail RB, Dellagi K (2005). The predictive validity of naturally acquired delayed-type hypersensitivity to leishmanin in resistance to Leishmania major-associated cutaneous leishmaniasis. J Infect Dis.

[29] Reithinger R, Dujardin JC, Louzir H, Pirmez C, Alexander B, Brooker S (2007). Cutaneous leishmaniasis. Lancet Infect Dis.

[30] Weigle KA, Santrich C, Martinez F, Valderrama L, Saravia NG (1993). Epidemiology of cutaneous leishmaniasis in Colombia: environmental and behavioral risk factors for infection, clinical manifestations, and pathogenicity. J Infect Dis.

[31] Pedrosa Fde A, Ximenes RA (2009). Sociodemographic and environmental risk factors for American cutaneous leishmaniasis (ACL) in the State of Alagoas, Brazil. Am J Trop Med Hyg.

[32] Armijos RX, Weigel MM, Izurieta R, Racines J, Zurita C, Herrera W, Vega M (1997). The epidemiology of cutaneous leishmaniasis in subtropical Ecuador. Trop Med Int Health.

[33] Oliveira F, Doumbia S, Anderson JM, Faye O, Diarra SS, Traore P, Cisse M, Camara G, Tall K, Coulibaly CA, Samake S, Sissoko I, Traore B, Diallo D, Keita S, Fairhurst RM, Valenzuela JG, Kamhawi S (2009). Discrepant prevalence and incidence of Leishmania infection between two neighboring villages in Central Mali based on Leishmanin skin test surveys. PLoS Negl Trop Dis.

[34] Desjeux P (2001). Worldwide increasing risk factors for leishmaniasis. Med Microbiol Immunol.

[35] Feliciangeli MD, Mazzarri MB, Campbell-Lendrum D, Maroli M, Maingon R (2003). Cutaneous leishmaniasis vector control perspectives using lambdacyhalothrin residual house spraying in El Ingenio, Miranda State, Venezuela. Trans R Soc Trop Med Hyg.

[36] Campbell-Lendrum D, Dujardin JP, Martinez E, Feliciangeli MD, Perez JE, Silans LN, Desjeux P (2001). Domestic and peridomestic transmission of American cutaneous leishmaniasis: changing epidemiological patterns present new control opportunities. Mem Inst Oswaldo Cruz.

[37] Chaves LF, Calzada JE, Rigg C, Valderrama A, Gottdenker NL, Saldana A (2013). Leishmaniasis sand fly vector density reduction is less marked in destitute housing after insecticide thermal fogging. Parasit Vectors.

[38] Mengistu G, Laskay T, Gemetchu T, Humber D, Ersamo M, Evans D, Teferedegn H, Phelouzat MA, Frommel D (1992). Cutaneous leishmaniasis in south-western Ethiopia: Ocholo revisited. Trans R Soc Trop Med Hyg.

[39] Bern C, Amann J, Haque R, Chowdhury R, Ali M, Kurkjian KM, Vaz L, Wagatsuma Y, Breiman RF, Secor WE, Maguire JH (2006). Loss of leishmanin skin test antigen sensitivity and potency in a longitudinal study of visceral leishmaniasis in Bangladesh. Am J Trop Med Hyg.

[40] Turgay N, Balcioglu IC, Toz SO, Ozbel Y, Jones SL (2010). Quantiferon-Leishmania as an epidemiological tool for evaluating the exposure to Leishmania infection. Am J Trop Med Hyg.

[41] Gidwani K, Rai M, Chakravarty J, Boelaert M, Sundar S (2009). Evaluation of leishmanin skin test in Indian visceral leishmaniasis. Am J Trop Med Hyg.

